# Unsatisfied

**Published:** 1890-02-08

**Authors:** 


### Words of Consolation.

### "UNSATISFIED."

*(For Reading to the Sick.)*

There is a natural desire in every man's heart for for something it has not got. He may have money, health, and happiness, yet deep down in his bosom lies a craving, which must in the end make itself felt. For man was made in the image of God, and much as that image may be defaced he can never be satisfied with anything short of God Himself. Let us see what are the things which we so earnestly desire, and which so often hide the true wants of the soul from our eyes. One man longs for wealth, another for more pleasure; this one for strength and ability to carry out some great work, that man for ease and indolence. Grant them their wishes, and before long the coveted thing has ceased to please, and a new object is ardently sought for. No doubt the prodigal son when his father gave him his portion of goods to do as he liked with felt that he should never again be discontented. He soon, however, ran through all the pleasures the world could afford, then wasted his substance in riotous living, and "began to be in want." Began, for at once followed poverty, sickness, starvation, which mean want in its saddest, sternest forms. Robbed of all that had been so dear to him before, deserted by the false friends who gathered round in his prosperity, he was reduced to tending swine, and was glad to eat even the husks with which those unclean animals were fed. His life now changes; he begins to come to himself. What a true picture this is of man's heart! In prosperity he is unmindful of God. He is content to forget all His mercies. But no sooner does misfortune or sickness fall on him than he sees how truly poor and naked and wretched and miserable he really is. What shall he do to deliver himself from his miserable plight? The prodigal solved the difficulty for himself and all other wanderers from the fold. He remembered the happiness of his father's hired servants, thinks about it, and makes a resolution. He says, "I will arise and go to my Father, and will say unto him, Father, I have sinned against heaven, and before thee, and am no more worthy to be called thy son."

You who are lying prostrate with suffering may know nothing of the excesses of the son in the parable, yet have you not forgotten to whom you owe everything, and have you not been equally willing to take your life into your own hands and please yourselves? Has it been a success? Have you been happy? Rather, do you not feel that without the care and protection of your Heavenly father you are the most miserable of men? Look into your hearts and see what are the real wants of your soul. Life and health and strength are what you need. Arise, then, with the prodigal, and make the same confession—"I have sinned." You are, indeed, unworthy to be called God's child; but "if we confess our sins, He is faithful and just to forgive us our sins, and to cleanse us from all unrighteousness." Can you not bring yourself to do this; are you afraid of the consequenees of your former conduct? See how the Father received the wanderer! While he was yet a great way off he came to meet him, and fell on his neck and kissed him; he did not reproach him, made no allusion even to the past. God can see in you the first signs of penitence, and will meet you more than half-way, for the love of our Heavenly Parient knows no bounds. Stretch out the arm of faith and prayer and He will grasp them; call upon him, for His ears are open to the faintest cry. Do not wait to try and make yourself fit, for

> "All the fitness He requireth
> Is to feel our need of Him."

